# Conserved cross-domain protein-to-mRNA ratios enable proteome prediction in microbes

**DOI:** 10.1128/mbio.01411-25

**Published:** 2025-07-24

**Authors:** Mengshi Zhang, Changyi Zhang, Anayancy Ramos, Rachel J. Whitaker, Marvin Whiteley

**Affiliations:** 1 School of Biological Sciences and Center for Microbial Dynamics and Infection, Georgia Institute of Technology 1372, Atlanta, Georgia, USA; 2 Emory-Children’s Cystic Fibrosis Center, Atlanta, Georgia, USA; 3 Carl R. Woese Institute for Genomic Biology, University of Illinois at Urbana-Champaign 14589, Urbana, Illinois, USA; Indiana University Bloomington, Bloomington, Indiana, USA

**Keywords:** *Pseudomonas aeruginosa*, RNA-to-protein conversion, transcriptomics, proteome, translation, microbiome functional inference, cross-domain prediction

## Abstract

Microbial communities are often studied by measuring gene expression (mRNA levels), but translating these data into functional insights is challenging because mRNA abundance does not always predict protein levels. Here, we present a strategy to bridge this gap by deriving gene-specific RNA-to-protein conversion factors that improve the prediction of protein abundance from transcriptomic data. Using paired mRNA–protein data sets from seven bacteria and one archaeon, we identified orthologous genes where mRNA levels poorly predicted protein abundance, yet each gene’s protein-to-RNA ratio was consistent across these diverse organisms. Applying the resulting conversion factors to mRNA levels dramatically improved protein abundance predictions, even when the conversion factors were obtained from distantly related species. Remarkably, conversion factors derived from bacteria also enhanced protein prediction in an archaeon, demonstrating the robustness of this approach. This cross-domain framework enables more accurate functional inference in microbiomes without requiring organism-specific proteomic data, offering a powerful new tool for microbial ecology, systems biology, and functional genomics.

**IMPORTANCE:**

Deciphering the biology of natural microbial communities is limited by the lack of functional data. While transcriptomics enables gene expression profiling, mRNA levels often fail to predict protein abundance, the primary indicator of microbial function. Prior studies addressed this by calculating RNA-to-protein (RTP) conversion factors using conserved protein-to-RNA (ptr) ratios across bacterial strains, but their cross-species and cross-domain utility remained unknown. We generated comprehensive transcriptomic and proteomic data sets from seven bacteria and one archaeon spanning diverse metabolisms and ecological niches. We identified orthologous genes with conserved ptr ratios, enabling the discovery of RTP conversion factors that significantly improved protein prediction from mRNA, even between distant species and domains. This reveals previously unrecognized conservation in ptr ratios across domains and eliminates the need for paired proteomic data in many cases. Our approach offers a broadly applicable framework to enhance functional prediction in microbiomes using only transcriptomic data.

## INTRODUCTION

A major challenge in understanding the biology of natural microbial communities is the lack of robust functional data for the microbes within these ecosystems. In many cases, this has hampered the development of biologically relevant *in vitro* and *in vivo* models (1–3) that can provide fundamental, functional insights into the biology of natural microbial communities. One way to tackle this problem is to quantify gene expression in native communities and use this data to infer microbial function. This transcriptomic approach has been highly successful in assessing the function of *in situ* microbial communities, including the human microbiome during health and disease (4–7), marine environments (8, 9), soil (10, 11), and even during space flight (12–14). Although RNA-seq has been widely used to study bacterial physiology *in situ*, a critical concern arises regarding whether mRNA levels accurately predict protein levels, which are the primary functional units of a cell (15, 16). While previous studies have established a positive correlation between mRNA and protein levels (17–31), for certain genes, mRNA is not a sufficient predictor of protein levels (32–34). However, performing comprehensive quantitative proteomics of microbes in natural ecosystems is incredibly challenging (35–38). Although this limitation may be overcome in the future via technological innovation (39–41), RNA-seq will remain an economical and powerful technique for assessing microbial function in complex environments. Thus, there is a pressing need to bridge the gap between mRNA levels and protein prediction.

To address this gap, large data sets have been generated to assess the protein-to-RNA ratio (ptr) of each gene, asking the question: Is the ptr highly conserved, or is it impacted by microbial strain and environmental factors? Surprisingly, these studies have shown that the ptr for some genes is highly conserved between bacterial strains in multiple growth environments and across a range of growth rates observed during human infection (31, 42–44). This conservation in ptr has allowed for the calculation of an RNA-to-protein (RTP) conversion factor for these genes, which is a gene-specific conversion factor that can be applied to mRNA measurements to improve prediction of protein levels (43, 45). Notably, RTP conversion factors are independent of growth rate in the bacterium *Pseudomonas aeruginosa* (43), which is critical as bacteria in natural environments often have heterogeneous growth rates. Thus, this approach has enhanced our understanding of microbial function in complex natural environments, providing a straightforward approach for correcting functional predictions in transcriptomic data.

While RTP conversion factors have the potential to greatly enhance our understanding of microbial function in natural ecosystems, their calculation requires large proteomic and transcriptomic data sets, which are not available for most microbes and can be costly to obtain. One potential solution is to use comprehensive data sets in a limited number of microbes to calculate RTP conversion factors that can be applied broadly to multiple microbes. The primary challenge to this approach is identifying orthologous genes in multiple microbes that have consistent ptr ratios. Here, we addressed this challenge by calculating ptr ratios for thousands of genes using comprehensive transcriptome and proteome data sets from Gram-negative bacteria, Gram-positive bacteria, and an archaeon. We discovered that the overall correlation of mRNA and protein is similar across diverse prokaryotes, with mRNA and protein positively correlated. In addition, we identified orthologous genes in which mRNA was not predictive of protein, but the associated ptr ratios for these genes were similar across the diverse microbes tested. Calculation of RTP conversion factors for these genes from highly related microbes significantly improved the accuracy of protein prediction from mRNA of distantly related microbes, indicating a core subset of genes in which RTP conversion factors can be broadly applied.

## RESULTS

To comprehensively investigate mRNA-protein correlations from a broad range of microbes, we performed simultaneous transcriptomics and proteomics of five bacterial species and one archaeon. The bacteria include two proteobacteria (*Pseudomonas aeruginosa* and *Aggregatibacter actinomycetemcomitans*), two Firmicutes (*Staphylococcus aureus* and *Streptococcus gordonii*), and one Bacteriodota (*Porphyromonas gingivalis*) (Table 1; Data sets S1 and S4). Among these microbes, three are commonly found in the human oral cavity (*A. actinomycetemcomitans*, *S. gordonii*, and *P. gingivalis*), one is a common member of the human microbiota and an opportunistic pathogen (*S. aureus*), and one is an opportunistic pathogen (*P. aeruginosa*). *Sulfolobus islandicus*, a hyperthermophilic, acidophilic archaeon belonging to the TACK superphylum, was also used (46, 47). These microbes display a wide spectrum of generation times, spanning from 20 minutes (*S. aureus*) to 10 hours (*S. islandicus*), and the genome sizes vary from 2.1 megabase pairs to 6.5 megabase pairs (Data set S1). In addition to these six species, we also included three strains of *A. actinomycetemcomitans* (624, VT1169, and Y4) with one strain grown in two different media (Table 1). This comprehensive data set allowed us to study mRNA-protein relationships within a bacterial species, between bacterial species, between bacteria and one archaeon, and across diverse growth conditions.

**TABLE 1:** Microbes used and growth conditions

Microbe	Strain	Media	Code
*P. aeruginosa*	PA14	Minimal (43)	*Pa*
*A. actinomycetemcomitans*	Y4	TSBYE	*Aa* Y4
*A. actinomycetemcomitans*	624	TSAYE	Aa 624
*A. actinomycetemcomitans*	VT1169	TSBYE	*Aa* VT1169_TY_
*A. actinomycetemcomitans*	VT1169	BHIYE	*Aa* VT1169_BY_
*P. gingivalis*	*Pg*	TSBYE	*Pg*
*S. aureus*	*Sa*	BHI	*Sa*
*S. gordonii*	*Sg*	TSBYE	*Sg*
*S. islandicus*	*Si*	DT media (48)	*Si*

### mRNA and protein levels are positively correlated

**Fig 1:**
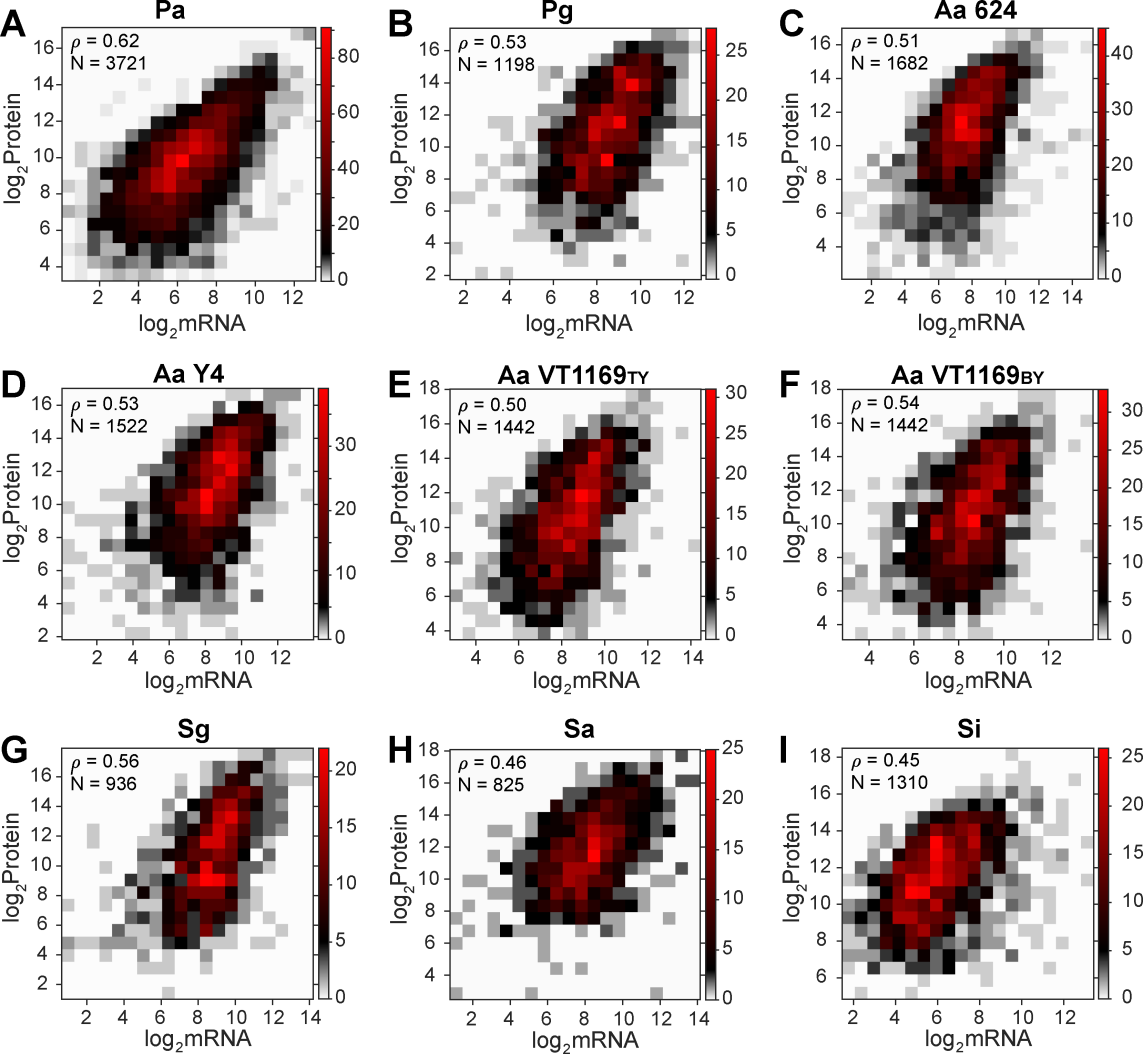
mRNA and protein levels are positively correlated in diverse prokaryotes. Binned scatterplots showing the relationship between protein abundances and mRNA abundances across a diverse range of prokaryotes, including Gram-negative bacteria (**A–F**), Gram-positive bacteria (**G and H**), and an archaeon (**I**). Spearman rank correlation coefficients (ρ) ranged from 0.45 to 0.62, demonstrating a moderate positive correlation between mRNA and protein abundances. These analyses included genes detected in both transcriptome and proteome data sets (number of genes indicated by N). The color scale on the right ordinate represents the number of genes at specific *x-y* coordinates.

We first assessed the correlation of mRNA and protein for each microbe, restricting our analysis to the genes in which both the mRNA and protein were detected (Data set S2). The number of genes satisfying this criterion varied between the microbes, with the highest percentage in *A. actinomycetemcomitans* (78% of total genes) and the lowest in *S. aureus* (30% of total genes) (Data sets S1 and S2). For all microbes, we quantified the correlation between protein and mRNA abundances by calculating the Spearman rank correlation coefficient (ρ) of the log2-transformed data. Positive correlations between mRNA and protein abundances were observed for all microbes with correlation coefficients ranging from 0.45 to 0.62 (Fig. 1; Fig. S1). The coefficient of determination (*R*^2^) between mRNA and protein abundances revealed that, on average, 27% (range of 18% to 38%) of the variability in protein levels can be explained by the variability in mRNA levels. The positive correlation between mRNA and protein levels observed here agrees with previous studies using both bacteria and eukaryotes (17–31).

### mRNA and protein abundances of essential genes are more highly correlated than non-essential genes during exponential growth

**Fig 2:**
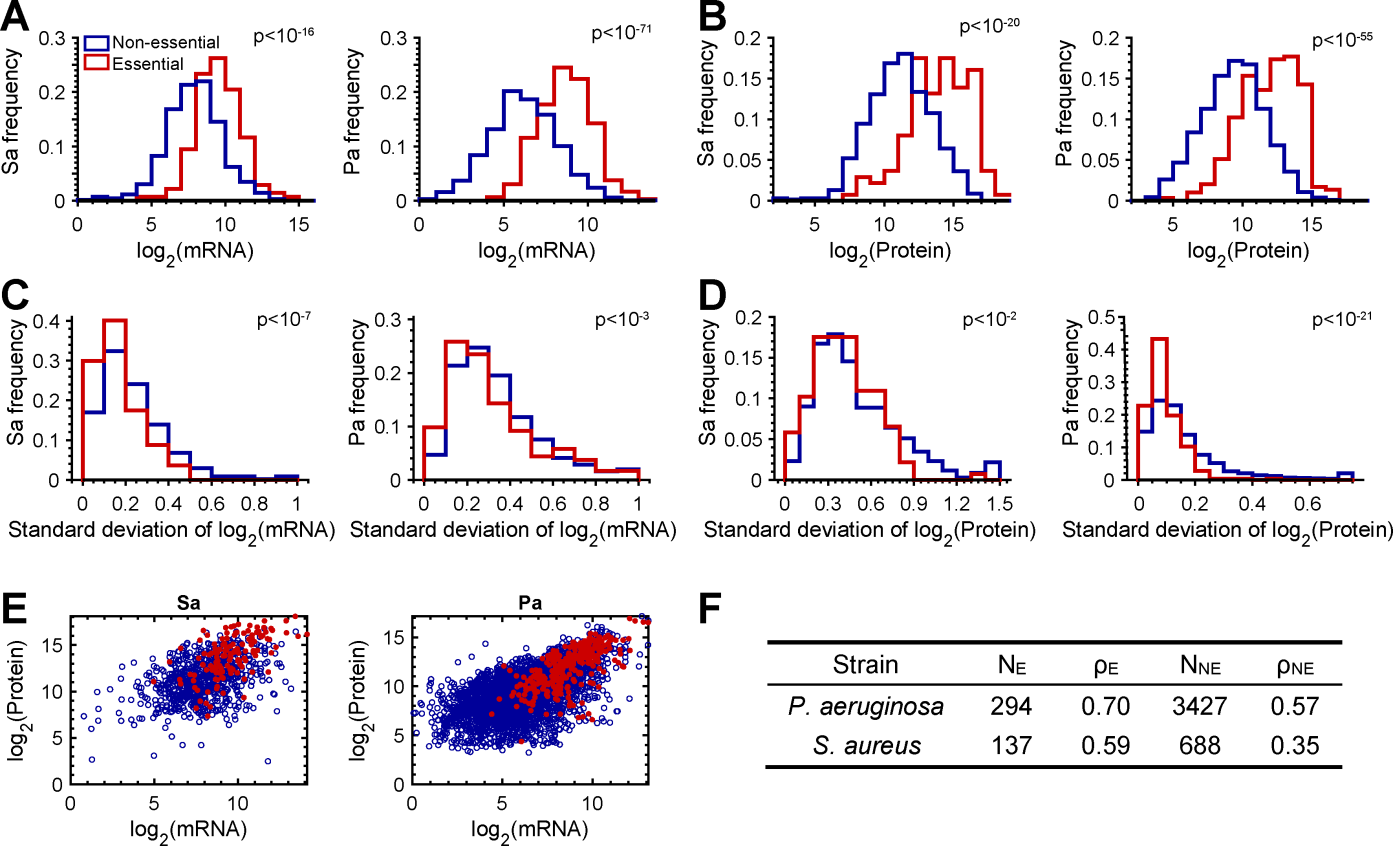
Essential genes are highlyexpressed, have lower variance, and increased mRNA-protein correlations in both *P. aeruginosa* and *S. aureus*. (**A and B**) Distributions of mRNA (**A**) and protein (**B**) abundances for *P. aeruginosa* and *S. aureus* essential and non-essential genes. Histograms were constructed with a bin size of 1 of log2-normalized mRNA and protein levels. (**C and D**) Distributions of the standard deviations of mRNA (**C**) and protein (**D**) abundances for *P. aeruginosa* and *S. aureus* essential and non-essential genes. Histograms of standard deviations were constructed with a bin size of 0.1 in log2-normalized mRNA and a bin size of 0.05 in log2-normalized protein levels. Standard deviations larger than the range shown in the abscissa are included in the largest bin. (**E**) Scatterplot of *P. aeruginosa* and *S. aureus* essential (red circles) and non-essential (blue circles) genes with associated Spearman rank correlation coefficient (ρ) for essential (ρ_E_) and non-essential (ρ_NE_) genes. All *P*-values were calculated using a Wilcoxon rank-sum test. (**F**) Spearman rank correlation coefficients for *P. aeruginosa* and *S. aureus* essential and non-essential genes. Notably, Pearson correlation also yields similar trends as Spearman rank correlations for *P. aeruginosa* (ρ_E, Pearson_ = 0.67, ρ_NE, Pearson_ = 0.58) and *S. aureus* (ρ_E, Pearson_ = 0.58, ρ_NE, Pearson_ = 0.37).

We previously reported that *P. aeruginosa* essential genes have (i) higher mRNA and protein abundances than non-essential genes; (ii) less variance in mRNA protein abundance; and (iii) a higher correlation of mRNA and protein than non-essential genes (43). We re-examined these findings with a more refined list of *P. aeruginosa* essential genes, identified through additional transposon sequencing data (49). This analysis identified 302 *P*. *aeruginosa* essential genes shared across diverse growth conditions, with 294 of these genes present in both our mRNA and protein data sets (49, 50). Examination of mRNA-protein abundances and correlations revealed that, as previously reported (43), *P. aeruginosa* essential genes are expressed at higher levels (Fig. 2A and B), have reduced variance (Fig. 2C and D), and have higher correlation coefficients compared to non-essential genes (Fig. 2E and F). Notably, this elevated correlation is not attributable to the differences in expression levels among essential and non-essential genes (Fig. S6).

To determine whether this phenomenon is restricted to *P. aeruginosa* essential genes, we next examined mRNA-protein abundances and correlations of both essential and non-essential *S. aureus* genes. We chose to focus on *S. aureus* because, like *P. aeruginosa*, the essential genome is well-defined from genome-wide transposon sequencing and RNA interference data (51, 52). In all, 302 essential genes were reported using RNA interference (53) and 351 genes were reported using transposon sequencing (54), with 166 genes shared between these two conditions. Of these 166 shared genes, we focused on 137 genes that were present in both our mRNA and protein data sets. As in *P. aeruginosa*, we discovered that (i) mRNA and protein abundances were higher for essential genes compared to non-essential genes (Fig. 2A and B); (ii) despite increased abundances of both mRNA and protein, essential genes had significantly lower variance compared to non-essential genes (Fig. 2C and D); and (iii) correlation between mRNA and protein levels of *S. aureus* essential genes was higher (0.59) than that of non-essential genes (0.35) (Fig. 2E and F). These data provide further evidence that mRNA and proteins from essential genes are produced at higher, and less variable levels than non-essential genes and have higher mRNA-protein correlations compared to non-essential genes (Fig. 2; Fig. S5).

### Protein levels of orthologous genes have a higher correlation than the corresponding mRNAs during exponential growth

**Fig 3:**
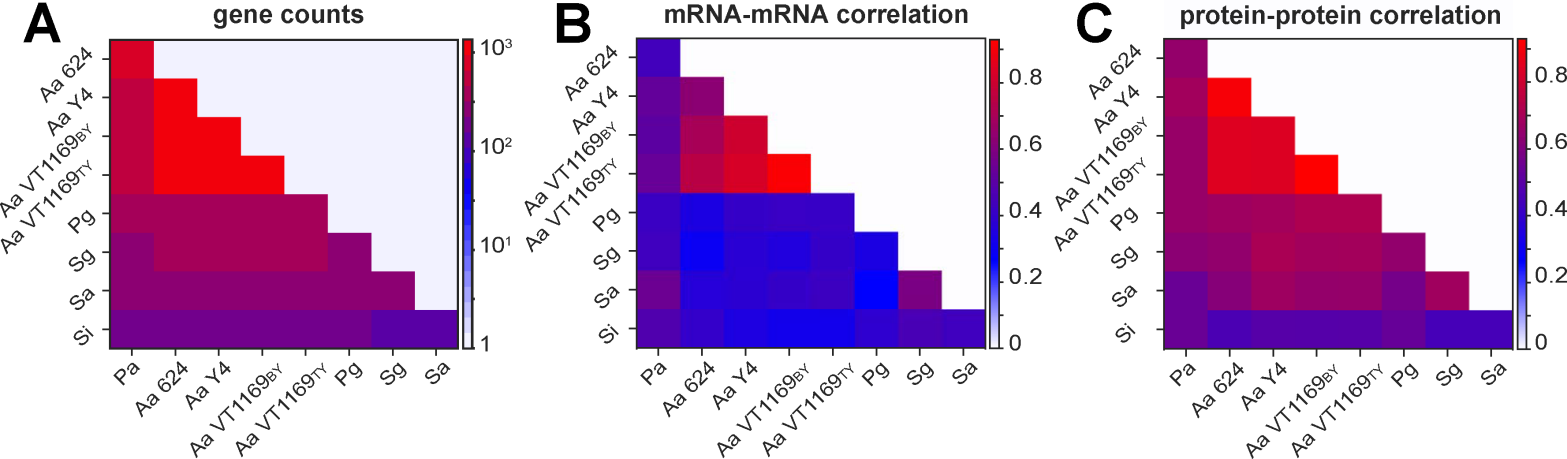
The correlation between orthologous protein abundances is higher than that of corresponding mRNA abundances. (**A**) Pairwise identification of the number of orthologous genes in the prokaryotes used in this study. Colors represent the number of orthologous genes between pairs as indicated on the right ordinate. (**B**) Pairwise Spearman rank correlation coefficients of the abundances of orthologous mRNAs. (**C**) Pairwise Spearman rank correlation coefficients of the abundances of orthologous proteins. For panels B and C, colors represent the correlation among each pairwise group as indicated on the right ordinates.

Previous studies in multiple eukaryotes have demonstrated a higher correlation in the abundance of orthologous proteins compared to their corresponding mRNA levels (55–57). To determine whether this was also observed in bacteria, we quantified protein-protein correlations by calculating the Spearman rank correlation of orthologous proteins within each pair of bacteria (Fig. 3C) and similarly assessed the rank correlations for their respective mRNAs (Fig. 3B). Of the 36 pairs considered, the correlation in protein abundances was greater than that of mRNAs in 34 cases (Fig. 3), highlighting a significantly higher conservation of protein abundances compared to the corresponding mRNAs (*P* < 10^−6^, Wilcoxon signed-rank test). One caveat of this analysis is that it includes data from three *A. actinomycetemcomitans* strains, which have higher correlations and more orthologous genes (Fig. 3A). We investigated whether the observed trend was still observed when including only one *A. actinomycetemcomitans* strain; the same trend was observed and was highly statistically significant in all cases (Fig. S2 *P* <10^−4^, Wilcoxon signed-rank test).

### Identification of genes that have extreme protein-to-mRNA ratios across bacteria

We next asked the question: are the genes in which mRNA is not predictive of protein levels unique in each prokaryote, or are they shared between the microbes? To answer this question, we first identified “outlier” genes that have extreme ptr ratios in each microbe (Data set S3). We did not utilize data from *S. islandicus* for this analysis as there were insufficient orthologs between this archaeon and bacteria for a robust evaluation. To identify outliers, we used standardized residuals, which calculate the extent of deviation of each gene from the best-fit regression line of the genome-wide mRNA and protein levels. We defined outlier genes as those in which the absolute values of ptr ratios were ≥2 standard deviations away from the linear regression line between mRNA and protein abundances. We then categorized these outlier genes into two groups based on the sign of standardized residuals: those with a positive sign indicate higher protein levels than expected (termed high ptr) and those with a negative sign indicate lower protein levels than expected (termed low ptr) (Data set S3). Significantly higher numbers of genes with low ptr ratios compared to high ptr ratios were identified in all microbes examined (Table 2, *P* = 0.002, Wilcoxon signed-rank test). Furthermore, the distribution of ptr ratios is “left-skewed,” with a notable prevalence of genes showing low ptr ratios over high ptr ratios (Fig. S3), aligning with our identification of outlier genes using standardized residuals (Table 2).

**TABLE 2:** Number of genes with high and low ptr ratios identified using standardized residuals^*a*^

Microbe	No. of low ptr genes	No. of high ptr genes
*Pa*	151	17
*Aa* Y4	70	4
Aa 624	71	0
*Aa* VT1169_TY_	45	10
*Aa* VT1169_BY_	43	14
*Pg*	39	7
*Sa*	23	8
*Sg*	20	9
*Si*	40	5

^*a*^ High ptr genes are defined by standardized residuals greater than or equal to 2, and low ptr genes are defined by standardized residuals less than or equal to −2.

**Fig 4:**
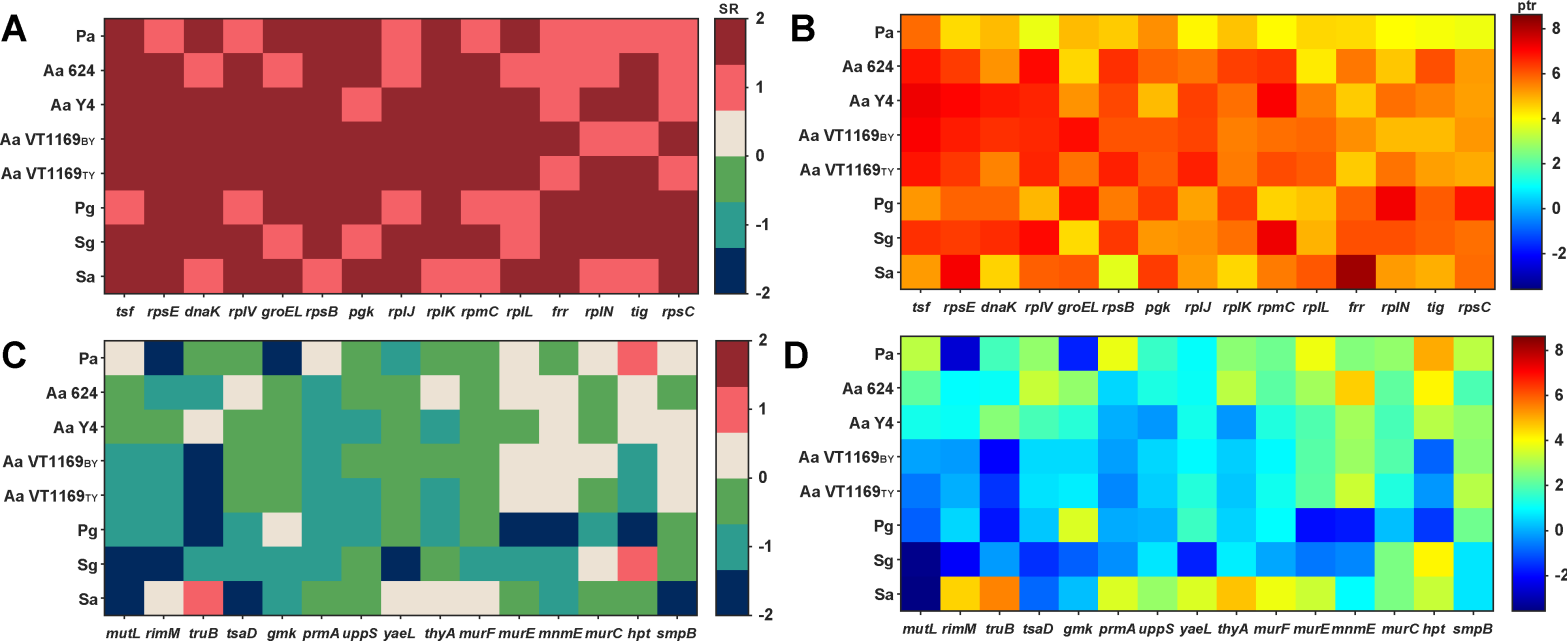
Some orthologous genes whose mRNAs were least predictive of protein levels are conserved among prokaryotes. (**A**) The top 15 orthologous genes ranked with the highest average standardized residuals across the microbes studied. Colors represents standardized residual for each microbe, and the scale is on the right ordinate. (**B**) ptr ratios of the top 15 orthologous genes. Colors represent ptr values, with the scale displayed on the right axis. (**C**) The bottom 15 orthologous genes ranked with the lowest average standardized residuals across the microbes studied. Colors represent standardized residual for each microbe, and the scale is on the right ordinate. (**D**) ptr ratios of the bottom 15 orthologous genes. Colors represent ptr values, and the scale is on the right ordinate.

To evaluate whether these orthologous outlier genes are bacterium-specific or shared among bacteria, we first averaged the standardized residuals for each orthologous outlier gene from all bacteria. When calculating this mean, we set the maximum standardized residual at an absolute value of 2 to limit the impact of single values on the mean. We then used this average standardized residual to rank all genes, then the top (Fig. 4A) and bottom (Fig. 4C) 15 genes were selected for further analysis. Examination of the variability in ptr ratios revealed that the top 15 outlier genes exhibited consistent and high ptr ratios among all bacteria (Fig. 4B), while the bottom 15 genes displayed more diverse ptr ratios (Fig. 4D). Notably, we identified one outlier gene, elongation factor Ts (tsf), that consistently displayed extremely high protein levels with moderate mRNA levels across all bacteria except *P. gingivalis* (Fig. 4A and B). These data reveal the existence of orthologous outlier genes that show similar ptr ratios among several, but not all, of the microbes tested.

### Using a gene-specific RNA-to-protein conversion factor to increase the predictability of protein from mRNA levels

Based on the lack of paired transcriptomic and proteomic data for most microbes, a primary goal of this work was to determine whether we can improve protein predictability from mRNA for a microbe of interest using RTP conversion factors calculated from other microbes. The basic approach is (i) to identify orthologous genes that are present in all, or a subset of the microbes; (ii) calculate an RTP conversion factor for these genes using data from one or a subset of microbes; and (iii) apply these RTP conversion factors to mRNA measurements from a microbe(s) not used in their calculation to determine whether they improve the ability to predict protein levels. While we previously showed that RTP conversion factors calculated from one *P. aeruginosa* strain can improve the mRNA-protein prediction in a second *P. aeruginosa* strain (42), whether this methodology would be successful using data from more distantly related microbes was unknown. To assess the robustness of this approach, we tested how RTP conversion factors improved mRNA-protein predictions within a bacterial species, between bacterial species, and between bacteria and archaea.

#### Within a bacterial species

**Fig 5:**
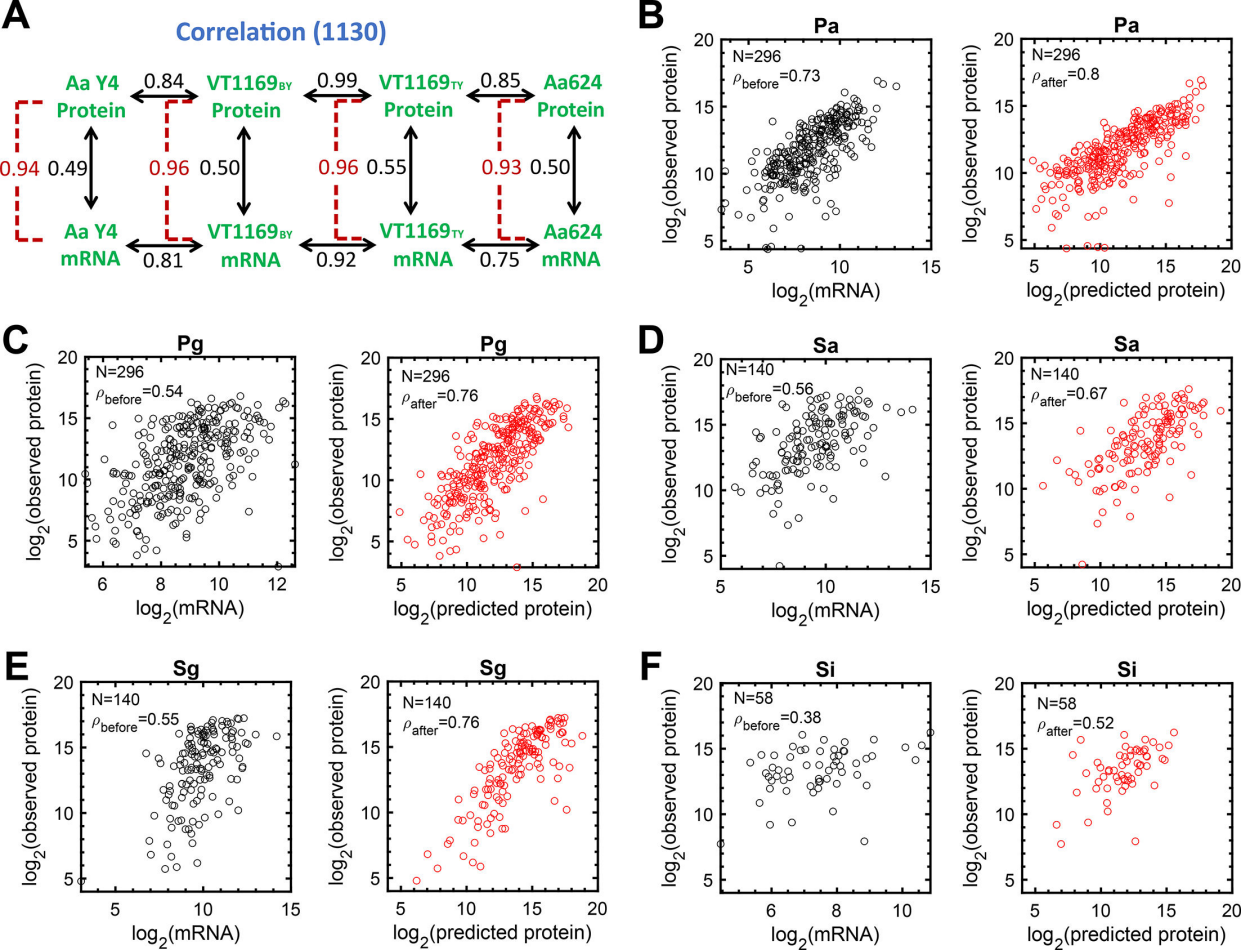
Improvement of predictivity of protein levels from respective mRNA abundances using gene-specific RTP conversion factors. (**A**) RTP conversion factors calculated from one strain of A. *actinomycetemcomitans* improve the predictivity of protein levels from mRNA abundances in other strains. Numbers are Spearman rank correlation coefficients for all shared orthologues before (black numbers) and after (red numbers) application of RTP conversion factors. For example, the mRNA-protein correlation coefficient of A. *actinomycetemcomitans* Y4 is 0.49 before and 0.94 after application of the RTP conversion factor, which was calculated using data from the other three A. *actinomycetemcomitans* data sets. Arrows indicate the samples that were correlated. (**B**) *P. aeruginosa* mRNA-protein correlation coefficients before (ρ_before_, left graph) and after (ρ_after_, right graph) application of the A. *actinomycetemcomitans* RTP conversion factors to orthologous genes. (**C**) *P. gingivalis* mRNA-protein correlation coefficients before (ρ_before_, left graph) and after (ρ_after_, right graph) application of the A. *actinomycetemcomitans* RTP conversion factors to orthologous genes. (**D**) *S. aureus* mRNA-protein correlation coefficients before (ρ_before_, left graph) and after (ρ_after_, right graph) application of the Gram-negative bacterial RTP conversion factors to orthologous genes. (**E**) *S. gordonii* mRNA-protein correlation coefficients before (ρ_before_, left graph) and after (ρ_after_, right graph) application of the Gram-negative bacterial RTP conversion factors to orthologous genes. (**F**) *S. islandicus* mRNA-protein correlation coefficients before (ρ_before_, left graph) and after (ρ_after_, right graph) application of the bacterial RTP conversion factors to orthologous genes. All improvements in correlation coefficients after applying the RTP conversion factors were statistically significant using the Wilcoxon signed-rank test (*P* < 0.002).

We first used the mRNA-protein data sets for three *A. actinomycetemcomitans* strains to assess how RTP conversion factors calculated for one strain improved the mRNA-protein correlation in other strains of the same species or the same strain grown in a different medium. These results demonstrated a significant increase in mRNA-protein correlation in all cases, with the correlation coefficient increasing on average from 0.51 (range: 0.49–0.55) to 0.95 (range: 0.93–0.96) (Fig. 5; Fig. S4). These results reveal that, similar to *P. aeruginosa* (43), *A. actinomycetemcomitans* RTP conversion factors calculated from one bacterial strain can significantly improve mRNA-protein correlations in other *A. actinomycetemcomitans* strains, even if different media are used for growth.

#### Within Gram-negative bacteria

We next used the mRNA-protein data sets from the three strains of *A. actinomycetemcomitans* to assess how application of the RTP conversion factors from this Gram-negative bacterium impacted mRNA-protein correlation in the Gram-negative bacteria *P. aeruginosa* and *P. gingivalis*. Application of the *A. actinomycetemcomitans* RTP conversion factors significantly improved mRNA-protein correlation coefficients for orthologous outlier genes in both bacteria, increasing from 0.73 to 0.80 for *P. aeruginosa* (Fig. 5B) and from 0.54 to 0.76 for *P. gingivalis* (Fig. 5C). These data indicate that RTP conversion factors calculated from a Gram-negative bacterium can improve protein prediction from mRNA for other Gram-negative bacteria.

#### Between Gram-negative and Gram-positive bacteria

Next, we assessed the impact of RTP conversion factors calculated from all the Gram-negative bacteria examined in this study (*A. actinomycetemcomitans, P. aeruginosa*, and *P. gingivalis*) on the mRNA-protein correlation coefficients in Gram-positive bacteria. The application of Gram-negative RTP conversion factors to the mRNA levels of Gram-positive bacteria resulted in substantial improvements in the mRNA-protein correlation coefficient for *S. aureus* from 0.56 to 0.67 (Fig. 5D) and *S. gordonii* from 0.55 to 0.76 (Fig. 5E). These data indicate that the RTP conversion factors from one bacterial group can improve protein-level predictivity from mRNA measurements for distantly-related bacteria.

#### Within all bacteria

To assess how RTP conversion factors calculated with data from a subset of bacteria impacted mRNA-protein correlation coefficients in other bacteria, we performed leave-one-out analysis. This approach involves excluding one bacterial data set from our RTP conversion factor calculations, then applying these conversion factors to the left-out transcriptome to determine the predictive accuracy of the observed protein levels. This approach consistently demonstrated that the correlations among the predicted and observed protein levels are significantly higher than the original mRNA-to-protein correlations (*P* = 0.0039, Wilcoxon signed-rank test, Table 3) under all conditions, further supporting the robustness of RTP conversion factors for improving protein prediction in bacteria.

**TABLE 3:** Leave-one-out analysis using bacterial RTP conversion factors^*h*^

Bacteria	Subset^*d*^ with *Aa* 624		Subset^*e*^ with *Aa* Y4		Subset^*f*^ with *Aa* VT1169_BY_		Subset^*g*^ with *Aa* VT1169_TY_	
	ρ_S_^*b*^	ρ_corrected_^*c*^	ρ_S_	ρ_corrected_	ρ_S_	ρ_corrected_	ρ_S_	ρ_corrected_
*N^a^*	144	144	142	142	142	142	142	142
*Pa*	0.74	0.82	0.73	0.82	0.74	0.83	0.73	0.83
*Aa^e^ (Aa* Y4)	–^*i*^	–	0.39	0.75	–	–	–	–
*Aa^d^ (Aa* 624)	0.54	0.76	–	–	–	–	–	–
*Aa^g^* (VT1169_TY_)	–	–	–	–	–	–	0.49	0.77
*Aa^f^* (VT1169_BY_)	–	–	–	–	0.41	0.74	–	–
*Pg*	0.50	0.74	0.50	0.76	0.49	0.77	0.49	0.77
*Sg*	0.55	0.75	0.55	0.79	0.54	0.76	0.54	0.76
*Sa*	0.58	0.71	0.57	0.70	0.57	0.71	0.57	0.71

^*a*^ *N* is the number of orthologous genes used.

^*b*^ ρ_S_, mRNA-protein correlation coefficient of orthologous genes before application of RTP conversion factors. RTP conversion factors were calculated for each bacterium using data from the other bacteria (e.g., for *Pa*, the *Pa* data were left out with only one *Aa* data set used).

^*c*^ ρ_corrected_, correlation coefficient after applying the RTP conversion factor.

^*d*^ Use *Aa* 624 to represent the *Aa* species. d-g presents possible subsets of bacteria with only one Aa strain present.

^*e*^ Use *Aa* Y4 to represent the *Aa* species.

^*f*^ Use *Aa* VT1169_BY_ to represent the *Aa* species.

^*g*^ Use *Aa* VT1169_TY_ to represent the *Aa* species.

^*h*^ All possible subsets of bacteria with only one *Aa* strain present. Gray shading is used to indicate different subsets of data and improve readability and organization.

^*i*^ –, not applicable.

#### Between a bacterium and an archaeon

To determine whether RTP conversion factors could be used to improve mRNA-protein predictivity across domains of life, we applied RTP conversion factors calculated with data from all bacteria to protein measurements from *S. islandicus*. These data revealed a significant improvement in protein prediction in *S. islandicus*, with an increased correlation coefficient of from 0.38 to 0.52 (Fig. 5F). It should be noted that the number of genes used in this comparison is low (*n* = 58), limited by the number of shared orthologous genes in bacteria and *S. islandicus*.

## DISCUSSION

The goal of this work was twofold: (i) to explore the mRNA-protein relationship across diverse microbes and (ii) to determine whether mRNA-protein ratios from a specific bacterium or a group of bacteria can be used to improve protein predictivity from mRNA levels in more distantly related genera. Our results reveal that RTP conversion factors, while limited to orthologous genes, are highly effective at improving the prediction of protein levels within a bacterial species, among bacterial species, and between bacteria and an archaeon. This approach has the potential to substantially improve our understanding of microbial function in natural environments where metatranscriptomic data exist, particularly for microbes that are difficult to culture or in ones in which paired proteomic-transcriptomic data do not exist. The data generated in this study will be particularly impactful in microbiomes dominated by bacteria that are highly related to those studied here, including the human oral microbiome and the microbiomes of chronically infected cystic fibrosis (CF) lungs and chronic wounds. In addition, while the *S. islandicus* paired transcriptome-proteome data provided a means of comparing the effectiveness of RTP conversion factors across domains of life, we anticipate that these data will have even greater utility for aiding in protein prediction from mRNA in highly related archaea. One caveat to our studies is that our comparisons were performed in exponentially growing cells, which may have distinct ptr ratios compared to non-growing (stationary phase) cells. Future studies are planned to address this possibility.

The data generated in this study also allowed us to test whether our previous observation (43), that *P. aeruginosa* essential genes are more predictive of protein levels than non-essential genes, is observed in other bacteria. Our discovery that essential gene mRNA is highly predictive of protein levels in *S. aureus* (Fig. 2) suggests that this phenomenon may be more widespread in bacteria. We focused on *S. aureus* for this analysis as the essential genome has been well-defined, and in the future, we plan to perform this analysis on the other bacteria in this study once their essential genomes are known. We did attempt to perform this analysis with a “core” bacterial essential genome, but there were too few genes in this list with sufficient transcriptome/proteome data for analysis. In addition to higher mRNA-protein correlations, *S. aureus* essential genes also had higher mRNA and protein levels than non-essential genes, consistent with studies in *P. aeruginosa* (43) and yeast and human cell lines (58, 59). Indeed, only 10 out of 294 genes in *P. aeruginosa* and 2 out of 137 genes in *S. aureus* showed mRNA levels below 2^6^ (Fig. 2E). As we previously discussed (43), we do not think that the high protein predictability of essential gene mRNA is due to detection bias, as non-essential genes with similar protein and mRNA levels have lower correlation (Fig. 2). Instead, it is likely that essential genes act as hubs in protein-protein interaction networks, similar to that observed in human cells (58), and are thus less impacted by post-transcriptional regulatory mechanisms.

Like many other studies, we found that mRNA and protein are generally positively correlated, with the highest Spearman rank correlation coefficients observed in *P. aeruginosa* and the lowest in *S. islandicus* (Fig. 1). Correlations were similar across domains, despite different metabolic capacities, genome size, and ecological niches. Our data complement the many studies in eukaryotes, spanning from unicellular yeast to multicellular organisms, including tissue-specific mRNA-protein correlations, that are within a similar range (Fig. 1). Similar to the improvement observed when using bacterial RTPs to improve protein predictivity in *S. islandicus*, the bacterial data generated in this study will likely not significantly impact protein predictivity from mRNA in eukaryotes, as the number of shared outlier orthologous genes will be minimal.

Among genes exhibiting low predictivity of protein levels (Table 2), a higher number displayed low protein levels and moderate/high mRNA levels (low ptr). However, the orthologous outlier genes most shared between all or a subset of microbes had high protein levels and moderate mRNA levels (high ptr). In addition, the ptr ratios of orthologous outlier genes revealed a narrower range of values (~100-fold) compared to those in each species (>1,000-fold). While the underlying mechanisms are not known, it is plausible that many of these genes are subject to post-transcriptional regulation, potentially through mRNA/protein degradation or the action of small regulatory RNAs. In addition to identifying genes that are likely post-transcriptionally regulated, ptr ratios for these genes may also provide insights into the mechanisms of regulation through analysis of the 5′ UTR sequences.

This work expands a previous framework developed by our laboratory in *P. aeruginosa* (43) and another laboratory in eukaryotes (45), utilizing RTP conversion factors to improve functional inferences from transcriptomics data. The finding that conversion factors calculated for orthologous genes from existing bacterial data sets can be used to improve protein predictivity provides the opportunity to re-evaluate bacterial metatranscriptomic data, ultimately deepening our understanding of microbial function *in situ*. This will require the acquisition of some minimal proteomic data from these natural communities for benchmarking, which can likely be performed in well-studied model systems, such as the oral and CF lung microbiomes (60, 61).

## MATERIALS AND METHODS

### Strains, media, and growth conditions

The strains, media, and growth conditions are listed in Table 1 and Data set S1. Cell culture growth was monitored by measuring the OD_600_ of 0.75 mL aliquots, and samples were collected for transcriptomic and proteomic analyses at an OD_600_ of 0.5–0.7. All *A. actinomycetemcomitans* strains were grown in either filter-sterilized tryptic soy broth (BD Difco) supplemented with 0.5% yeast extract (Fluka) (TSBYE), shaking at 165 rpm, Brain Heart Infusion with 0.5% yeast extract (BHIYE), shaking at 165 rpm, or on membranes on the surface of tryptic soy agar plates (BD Difco) supplemented with 0.5% yeast (TSAYE) as described (62, 63) in a 5% CO_2_ atmosphere at 37°C. *S. gordonii* was grown in TSBYE in a 5% CO_2_ atmosphere at 37°C without shaking. *P. gingivalis* was grown anaerobically in TSBYE broth +5 µg/mL hemin +1 µg/mL menadione. Anaerobic growth was achieved in an anaerobic chamber (Coy) in an 85% N_2_, 10% CO_2_, 5% H_2_ atmosphere at 37°C with shaking at 100 rpm. *S. aureus* was grown in Brain Heart Infusion (BHI) broth. *P. aeruginosa* was grown in chemostats as described (43, 64, 65) using the defined minimal medium MOPS-succinate (64, 66). The hyperthermophilic archaeon *S. islandicus* M.16.4 was cultivated aerobically in Tissue Culture Flasks (Fisher Scientific, USA) at 76°C–78°C and pH 3.5 without shaking in DT medium (48), which is comprised of a basal salt medium supplemented with 0.2% (wt/vol) Dextrin (Sigma-Aldrich, USA) and 0.1% (wt/vol) Ezmix N-Z-Amine A (Sigma-Aldrich, USA). Exponentially growing cells were diluted to an OD_600_ of 0.01 in a 130 mL DT medium, and their growth was monitored over an 8-day incubation period by measuring OD_600_.

### RNA-seq

For all microbes except *S. islandicus*, a 5 mL sample of bacterial culture was collected and mixed with 20 mL RNAlater, which was incubated at 4°C for less than 24 h and then stored at −80°C. All samples were performed with four biological replicates. RNA-seq libraries were prepared as previously described for *in vitro* samples (43). For *S. islandicus*, ~2 × 10^9^ cells in exponential phase were collected by centrifugation at 2,600 × *g* for 20 min at 4°C. The resulting cell pellets were flash-frozen in a mixture of dry ice and absolute ethanol and stored at −80°C. Total RNA extraction was performed using the *mir*Van miRNA Isolation Kit (Invitrogen, USA), followed by DNase I (Thermo Fisher Scientific, USA) treatment, and RNA recovery using an RNA Clean & Concentrator-25 Kit (Zymo Research, USA), according to the manufacturer’s instructions. The concentration of RNA was quantified using the Qubit RNA HS Assay Kit (Invitrogen, USA). The constructed RNA-seq libraries were sequenced on an Illumina NextSeq 500 in the Molecular Evolution Core using 75 bp single-end runs at the Georgia Institute of Technology.

### Bioinformatic analysis of RNA-seq data

All RNA-seq reads were trimmed by cutadapt (version 3.2 with Python 3.8.6), reads with a minimum length of 22 bp (3) were then mapped using bowtie2 (version 2.4.2), and analyzed as previously described by the Whiteley group (1, 3, 4). Gene counts were tallied to the coding sequences (CDS) using the R package featureCounts (Subread v1.6.3) using the strand-specific option. For correlational analyses, raw data were normalized using Reads per million mapped reads (RPM). Four genes in *Si* were discarded from analysis as each had greater than 0.1 million reads on average, which were considered PCR hotspots. All figures were produced in MATLAB using the functions histogram, scatter, plot, imagesc, and PlotPub, and all analysis scripts are available at https://github.com/ZHANG0MS/mRNA-protein-relationships.

### Proteomics

Five milliliters of culture was collected on ice, centrifuged at 4°C at 14,000 *× g* for 5 min, and then washed once in 1 mL of ice-cold sterile PBS buffer. Cell pellets were stored at −80°C. Samples were then processed by the Emory Integrated Proteomics Core at Emory University. Samples were prepared for proteomics using a modified EasyPep digestion protocol (ThermoFisher). Briefly, pellets were suspended in 200 µL of EasyPep lysis buffer with Universal Nuclease (Thermo Scientific Pierce), followed by 3 rounds of sonication on ice (5 min in total). An aliquot (40 µg) of each sample was reduced and alkylated using 10 mM tris(2-carboxyethyl)phosphine (Thermo Scientific Pierce) and 40 mM chloroacetamide. Samples were incubated at 95°C for 10 min and then cooled to room temperature. Digestion was carried out overnight with 4 µg Trypsin/Lys-C Protease Mix (Thermo Scientific Pierce). Samples were desalted with EasyPep cleanup columns following the EasyPep clean-up protocol (ThermoFisher Cat# A40006) and dried down using a SpeedVac concentrator (LabConco). For each sample, peptides were labeled with TMTPro isobaric tags (ThermoFisher Cat# A44520) as previously described (67). High pH fractionation was performed based on a slightly modified protocol described here (68).

LC-MS/MS was carried out on a Dionex Ultimate 3000 rapid separation LC(RSLC) nano system (Thermo Fisher Scientific) and Lumos Orbitrap mass spectrometer (Thermo Fisher Scientific). Peptides were separated on an in-house assembled capillary column (15 cm ×150 µm internal diameter, Waters, Cat# 186008814) packed with 1.9 mm Reprosil-Pur C18 beads (Dr. Maisch, Ammerbuch, Germany) and eluted over a 20 min gradient using a Dionex Ultimate 3000 rapid separation LC (RSLC)nano system (Thermo Fisher Scientific). Mass spectrometry was performed with a high-field asymmetric waveform ion mobility spectrometry (FAIMS) pro equipped Orbitrap Eclipse mass spectrometer (Thermo Fisher Scientific) in positive ion mode using data-dependent acquisition with 2 × 1.5 second top speed cycles. Each cycle consisted of one full MS scan followed by as many MS/MS events that could fit within the given 1.5 second cycle time limit. MS scans were collected at a resolution of 120,000 (410–1,600 *m*/*z* range, 4 × 10^5^ AGC, 50 ms maximum ion injection time, FAIMS compensation voltage of −45 and −65). All higher energy collision-induced dissociation (HCD) MS/MS spectra were acquired at a resolution of 30,000 (0.7 *m*/*z* isolation width, 38% collision energy, 100% AGC target, 54 ms maximum ion time, TurboTMT on). Dynamic exclusion was set to exclude previously sequenced peaks for 30 seconds within a 10-ppm isolation window.

### Bioinformatic analysis of proteomics data

Raw mass spectrometry data files were processed with Proteome Discoverer (Thermo Fisher Scientific v3.0) and the search engine Sequest HT. The spectra were searched against the proteome database available in Uniprot or NCBI (Data set S1, sheet 3), and a contaminant database called the common repository of adventitious proteins (cRAP release 2018). The data were searched with MS peptide tolerances of 20 ppm and MS/MS tolerance for identification of 0.05 Da precursor. Quantification was achieved by calculating the sum of the most confident centroided reporter ions within a tolerance of 20 ppm. Trypsin was selected as the enzyme, and two missed cleavage sites were allowed. Database searches were performed with the following modifications: static modifications: carbamidomethylation of cysteine; variable modifications: oxidation of methionine, acetylation of N-terminal protein, loss of methylation of methionine, loss of methylation and acetylation of methionine. Peptides with a minimum length of six amino acids and labeled as “IsMasterProtein” were considered for identification. Proteins were only considered identified when observed in four replicates of a sample group. Quantitative values were normalized based on the total peptide intensity of the samples, ensuring all channels have the same total peptide group abundance. The results were then exported to Microsoft Excel for further data interpretation and statistical analysis. To enable direct comparisons across diverse species and different mass spectrometry runs, we developed species-specific scaling factors based on the ratio between each sample’s total protein abundance and the median abundance across all species, facilitating downstream correlation analyses. The results were then exported to Microsoft Excel for further data interpretation and statistical analysis. To mitigate the detection bias induced by different MS runs, we have used species-specific scale factors for all raw protein abundance data across MS runs and ensured standardized comparison of correlations across diverse species.

### Identification of orthologous genes

To identify orthologous genes, we performed orthology analysis using OrthoFinder2 (version 2.5.4) (69, 70). The protein sequences for all prokaryotes (Data set S1, sheet 3) were included in the same folder as the input for OrthoFinder. Multiple Sequence Alignment (MSA) was chosen as the orthology inference method. BLASTP was chosen as the search method to identify orthologs (71, 72), which increases accuracy by 1%–2% over the default method DIAMOND, and runtimes were optimized using Georgia Tech Partnership for an Advanced Computing Environment (PACE). The output files with protein accessions were then exported to Matlab for further data analysis.

### Pairwise correlation analysis among all bacteria

To calculate pairwise mRNA and protein correlations, orthologous genes were identified in which both mRNA and protein were detected by RNA-seq/proteomics. The mRNA-mRNA and protein-protein correlation between each pair was then assessed using Spearman rank correlation. Graphs were created in Matlab using the “imagesc” function, and the color bar was generated by the “cptcmap-pkg” function.

### Outlier analysis

We first fitted a linear regression model to the log2-transformed proteomic and transcriptomic data for each species, and then calculated standardized residuals for all genes. Outlier genes were defined as having an absolute standardized residual ≥2. Genes from each microbe were then placed into distinct bins based on their standardized residual values. To identify outlier genes shared across all bacteria, we focused on the 140 orthologous genes in bacteria and arranged them based on associated mean standardized residual values. The 15 genes with the highest and lowest mean standardized residuals were then used for further analysis.

### RTP conversion factor calculation and leave-one-out validation and subsampling methods

To predict protein levels from mRNA levels, we identify conditions for both training (${RTP}_{j}$) and testing data sets ($P_{i,j}^{pred}$) and their associated number of orthologous genes, and (i) calculated RTP conversion factors from specific groups of microbes; (ii) performed leave-one-out analysis for all bacterial data sets.

The gene-specific RTP conversion factor was calculated as described in the formula:


$${RTP}_{j}=\frac{\sum_{i=1}^{N=species}P_{i,j}-R_{i,j}}{N}\;for\;gene\;j$$

The calculation of the predicted protein level is described as follows:


$$P_{i,j}^{pred}={RTP}_{j}+R_{i,j}\;for\;gene\;j$$

To perform leave-one-out analysis, we calculated the left-out conversion factor (LF) as follows:


$${LF}_{j}=\frac{\sum_{i=1}^{N=species-1}P_{i,j}-R_{i,j}}{N-1}\;for\;gene\;j$$

$P_{i,j}$ and $R_{i,j}$ represent the log2-transformed protein and mRNA levels for orthologous gene j and species i. *j* equals the number of orthologous genes present in the training data sets, and *i* denotes the number of species included in the training data set. For instance, the calculation of bacterial RTP conversion factors ${RTP}_{j}$ involves 140 orthologous genes (*j* = 1, 2, …., 140) and 8 conditions (*N* = 8), including all Gram-positive and Gram-negative bacteria. When applying bacterial RTP conversion factors to archaea, we focused on 58 orthologous genes present in both bacteria and archaea (*j* = 1, 2, …., 58).

For leave-one-out analysis of bacterial data, the calculation of left-out conversion factors ${LF}_{j}$ involves the same number of orthologous genes (*N*_gene_ = 140) but with different combinations of all possible 7 out of 8 conditions by leaving one species out each time. When utilizing left-out conversion factors to predict for the left-out transcriptome, we focused on 140 orthologous genes present in all bacteria to predict for the left-out transcriptome (*j* = 1, 2, …., 140).

To perform subsampling and exclude the effects caused by stochastic events, we calculated the randomized conversion factor as described in the formula:


$${RTP}_{j}^{random}=\frac{\sum_{i=1}^{N=species}P_{i,k}-R_{i,h}}{N}\;for\;gene\;j$$

$P_{i,k}$ denotes the log2-transformed protein level of a randomized non-orthologous gene k in species *i*, while $R_{i,h}$ represents the log2-transformed mRNA levels of a non-orthologous gene h in species *i*. Briefly, the calculation of ${RTP}_{j}^{random}$for gene j involved randomly selecting a protein level of non-orthologous gene k and mRNA levels of gene h. Since the combination of extremely high protein levels and low mRNA levels does not exist, we restricted the degrees of freedom and avoided some combinations of mRNA and protein levels. Thus, we limited our selection of genes with paired mRNA and protein levels (k = h) to maintain a biologically relevant ptr ratio from each species.

To quantify the changes before and after applying the conversion factors, we calculated Spearman rank correlation between the observed mRNA and protein levels ρ_observed_ and correlation among the predicted proteins and observed proteins ρ_corrected_. The significance levels were determined using a paired non-parametric Wilcoxon signed-rank test.

## Data Availability

RNA-seq raw data are available in the NCBI Sequence Read Archive under BioProject PRJNA1152499, and proteomics raw data are deposited to the Mass Spectrometry Interactive Virtual Environment (MassIVE) database (accession MSV000095802) and also to ProteomeXchange (PXD055700).

## SUPPLEMENTAL MATERIAL

Data set S1 mbio.01411-25-s0001.xlsx List of microbes used in this study along with growth conditions, strain information, and reference genomes.

Data set S2 mbio.01411-25-s0002.xlsx The raw abundance values (log2 transformed) of mRNA and protein levels for each microbe used in this study.

Data set S3 mbio.01411-25-s0003.xlsx Protein-to-mRNA ratios for all microbes used in this study

Data set S4 mbio.01411-25-s0004.xlsx The raw abundance values of paired data sets with both gene and protein detected for each microbe used in this study.

Supplemental figures mbio.01411-25-s0005.pdf Figures S1 to S6.

ASM does not own the copyrights to Supplemental Material that may be linked to, or accessed through, an article. The authors have granted ASM a non-exclusive, world-wide license to publish the Supplemental Material files. Please contact the corresponding author directly for reuse.

## References

[1] Cornforth DM, Diggle FL, Melvin JA, Bomberger JM, Whiteley M. 2020. Quantitative framework for model evaluation in microbiology research using Pseudomonas aeruginosa and cystic fibrosis infection as a test case. mBio 11:e03042-19. doi:10.1128/mBio.03042-19 31937646 PMC6960289

[2] Lewin GR, Kapur A, Cornforth DM, Duncan RP, Diggle FL, Moustafa DA, Harrison SA, Skaar EP, Chazin WJ, Goldberg JB, Bomberger JM, Whiteley M. 2023. Application of a quantitative framework to improve the accuracy of a bacterial infection model. Proc Natl Acad Sci USA 120:e2221542120. doi:10.1073/pnas.2221542120 37126703 PMC10175807

[3] Lewin GR, Stocke KS, Lamont RJ, Whiteley M. 2022. A quantitative framework reveals traditional laboratory growth is a highly accurate model of human oral infection. Proc Natl Acad Sci USA 119:e2116637119. doi:10.1073/pnas.2116637119 34992142 PMC8764681

[4] Cornforth DM, Dees JL, Ibberson CB, Huse HK, Mathiesen IH, Kirketerp-Møller K, Wolcott RD, Rumbaugh KP, Bjarnsholt T, Whiteley M. 2018. Pseudomonas aeruginosa transcriptome during human infection. Proc Natl Acad Sci USA 115:E5125–E5134. doi:10.1073/pnas.1717525115 29760087 PMC5984494

[5] Duran-Pinedo A, Solbiati J, Teles F, Teles R, Zang Y, Frias-Lopez J. 2021. Long-term dynamics of the human oral microbiome during clinical disease progression. BMC Biol 19:240. doi:10.1186/s12915-021-01169-z 34742306 PMC8572441

[6] Duran-Pinedo AE, Chen T, Teles R, Starr JR, Wang X, Krishnan K, Frias-Lopez J. 2014. Community-wide transcriptome of the oral microbiome in subjects with and without periodontitis. ISME J 8:1659–1672. doi:10.1038/ismej.2014.23 24599074 PMC4817619

[7] Duran-Pinedo AE, Solbiati J, Teles F, Frias-Lopez J. 2023. Subgingival host-microbiome metatranscriptomic changes following scaling and root planing in grade II/III periodontitis. J Clin Periodontol 50:316–330. doi:10.1111/jcpe.13737 36281629 PMC13295556

[8] Shi Y, Tyson GW, DeLong EF. 2009. Metatranscriptomics reveals unique microbial small RNAs in the ocean’s water column. Nature 459:266–269. doi:10.1038/nature08055 19444216

[9] Frias-Lopez J, Shi Y, Tyson GW, Coleman ML, Schuster SC, Chisholm SW, Delong EF. 2008. Microbial community gene expression in ocean surface waters. Proc Natl Acad Sci USA 105:3805–3810. doi:10.1073/pnas.0708897105 18316740 PMC2268829

[10] Nuccio EE, Nguyen NH, Nunes da Rocha U, Mayali X, Bougoure J, Weber PK, Brodie E, Firestone M, Pett-Ridge J. 2021. Community RNA-Seq: multi-kingdom responses to living versus decaying roots in soil. ISME Commun 1:72. doi:10.1038/s43705-021-00059-3 36765158 PMC9723751

[11] Bang-Andreasen T, Anwar MZ, Lanzén A, Kjøller R, Rønn R, Ekelund F, Jacobsen CS. 2020. Total RNA sequencing reveals multilevel microbial community changes and functional responses to wood ash application in agricultural and forest soil. FEMS Microbiol Ecol 96:fiaa016. doi:10.1093/femsec/fiaa016 32009159 PMC7028008

[12] Zhao X, Yu Y, Zhang X, Huang B, Xu C, Zhang B, Bai P, Liu C. 2022. Phenotypic, genomic, and transcriptomic changes in an Acinetobacter baumannii strain after spaceflight in China’s Tiangong-2 space laboratory. Braz J Microbiol 53:1447–1464. doi:10.1007/s42770-022-00772-8 35763257 PMC9433479

[13] Ott E, Kawaguchi Y, Kölbl D, Rabbow E, Rettberg P, Mora M, Moissl-Eichinger C, Weckwerth W, Yamagishi A, Milojevic T. 2020. Molecular repertoire of Deinococcus radiodurans after 1 year of exposure outside the International Space Station within the Tanpopo mission. Microbiome 8:150. doi:10.1186/s40168-020-00927-5 33121542 PMC7597052

[14] Crabbé A, Schurr MJ, Monsieurs P, Morici L, Schurr J, Wilson JW, Ott CM, Tsaprailis G, Pierson DL, Stefanyshyn-Piper H, Nickerson CA. 2011. Transcriptional and proteomic responses of Pseudomonas aeruginosa PAO1 to spaceflight conditions involve Hfq regulation and reveal a role for oxygen. Appl Environ Microbiol 77:1221–1230. doi:10.1128/AEM.01582-10 21169425 PMC3067220

[15] Liang X, Martyniuk CJ, Simmons DBD. 2020. Are we forgetting the “proteomics” in multi-omics ecotoxicology? Comp Biochem Physiol Part D Genomics Proteomics 36:100751. doi:10.1016/j.cbd.2020.100751 33142247

[16] Jungblut PR, Hecker M. 2007. Proteomics of microbial pathogens. Wiley‐VCH Verlag GmbH & Co. KGaA.

[17] Becker K, Bluhm A, Casas-Vila N, Dinges N, Dejung M, Sayols S, Kreutz C, Roignant J-Y, Butter F, Legewie S. 2018. Quantifying post-transcriptional regulation in the development of Drosophila melanogaster. Nat Commun 9:4970. doi:10.1038/s41467-018-07455-9 30478415 PMC6255845

[18] Caglar MU, Houser JR, Barnhart CS, Boutz DR, Carroll SM, Dasgupta A, Lenoir WF, Smith BL, Sridhara V, Sydykova DK, Vander Wood D, Marx CJ, Marcotte EM, Barrick JE, Wilke CO. 2017. The E. coli molecular phenotype under different growth conditions. Sci Rep 7:45303. doi:10.1038/srep45303 28417974 PMC5394689

[19] Chen W-H, van Noort V, Lluch-Senar M, Hennrich ML, Wodke JAH, Yus E, Alibés A, Roma G, Mende DR, Pesavento C, Typas A, Gavin A-C, Serrano L, Bork P. 2016. Integration of multi-omics data of a genome-reduced bacterium: prevalence of post-transcriptional regulation and its correlation with protein abundances. Nucleic Acids Res 44:1192–1202. doi:10.1093/nar/gkw004 26773059 PMC4756857

[20] Choi YW, Park SA, Lee HW, Kim DS, Lee NG. 2008. Analysis of growth phase-dependent proteome profiles reveals differential regulation of mRNA and protein in Helicobacter pylori. Proteomics 8:2665–2675. doi:10.1002/pmic.200700689 18546151

[21] Corbin RW, Paliy O, Yang F, Shabanowitz J, Platt M, Lyons CE Jr, Root K, McAuliffe J, Jordan MI, Kustu S, Soupene E, Hunt DF. 2003. Toward a protein profile of Escherichia coli: comparison to its transcription profile. Proc Natl Acad Sci USA 100:9232–9237. doi:10.1073/pnas.1533294100 12878731 PMC170901

[22] Edfors F, Danielsson F, Hallström BM, Käll L, Lundberg E, Pontén F, Forsström B, Uhlén M. 2016. Gene‐specific correlation of RNA and protein levels in human cells and tissues. Mol Syst Biol 12:883. doi:10.15252/msb.20167144 27951527 PMC5081484

[23] Gygi SP, Rochon Y, Franza BR, Aebersold R. 1999. Correlation between protein and mRNA abundance in yeast. Mol Cell Biol 19:1720–1730. doi:10.1128/MCB.19.3.1720 10022859 PMC83965

[24] Jayapal KP, Philp RJ, Kok Y-J, Yap MGS, Sherman DH, Griffin TJ, Hu W-S. 2008. Uncovering genes with divergent mRNA-protein dynamics in Streptomyces coelicolor. PLoS One 3:e2097. doi:10.1371/journal.pone.0002097 18461186 PMC2367054

[25] Jeacock L, Faria J, Horn D. 2018. Codon usage bias controls mRNA and protein abundance in trypanosomatids. Elife 7:e32496. doi:10.7554/eLife.32496 29543155 PMC5896881

[26] Lu P, Vogel C, Wang R, Yao X, Marcotte EM. 2007. Absolute protein expression profiling estimates the relative contributions of transcriptional and translational regulation. Nat Biotechnol 25:117–124. doi:10.1038/nbt1270 17187058

[27] Maier T, Schmidt A, Güell M, Kühner S, Gavin A-C, Aebersold R, Serrano L. 2011. Quantification of mRNA and protein and integration with protein turnover in a bacterium. Mol Syst Biol 7:511. doi:10.1038/msb.2011.38 21772259 PMC3159969

[28] Marguerat S, Schmidt A, Codlin S, Chen W, Aebersold R, Bähler J. 2012. Quantitative analysis of fission yeast transcriptomes and proteomes in proliferating and quiescent cells. Cell 151:671–683. doi:10.1016/j.cell.2012.09.019 23101633 PMC3482660

[29] Ponnala L, Wang Y, Sun Q, van Wijk KJ. 2014. Correlation of mRNA and protein abundance in the developing maize leaf. Plant J 78:424–440. doi:10.1111/tpj.12482 24547885

[30] Riba A, Di Nanni N, Mittal N, Arhné E, Schmidt A, Zavolan M. 2019. Protein synthesis rates and ribosome occupancies reveal determinants of translation elongation rates. Proc Natl Acad Sci USA 116:15023–15032. doi:10.1073/pnas.1817299116 31292258 PMC6660795

[31] Wilhelm M, Schlegl J, Hahne H, Gholami AM, Lieberenz M, Savitski MM, Ziegler E, Butzmann L, Gessulat S, Marx H, Mathieson T, Lemeer S, Schnatbaum K, Reimer U, Wenschuh H, Mollenhauer M, Slotta-Huspenina J, Boese J-H, Bantscheff M, Gerstmair A, Faerber F, Kuster B. 2014. Mass-spectrometry-based draft of the human proteome. Nature 509:582–587. doi:10.1038/nature13319 24870543

[32] Maier T, Güell M, Serrano L. 2009. Correlation of mRNA and protein in complex biological samples. FEBS Lett 583:3966–3973. doi:10.1016/j.febslet.2009.10.036 19850042

[33] Liu Y, Beyer A, Aebersold R. 2016. On the dependency of cellular protein levels on mRNA abundance. Cell 165:535–550. doi:10.1016/j.cell.2016.03.014 27104977

[34] Buccitelli C, Selbach M. 2020. mRNAs, proteins and the emerging principles of gene expression control. Nat Rev Genet 21:630–644. doi:10.1038/s41576-020-0258-4 32709985

[35] Zhang Y, Fonslow BR, Shan B, Baek M-C, Yates JR III. 2013. Protein analysis by shotgun/bottom-up proteomics. Chem Rev 113:2343–2394. doi:10.1021/cr3003533 23438204 PMC3751594

[36] Bantscheff M, Lemeer S, Savitski MM, Kuster B. 2012. Quantitative mass spectrometry in proteomics: critical review update from 2007 to the present. Anal Bioanal Chem 404:939–965. doi:10.1007/s00216-012-6203-4 22772140

[37] Liessi N, Pedemonte N, Armirotti A, Braccia C. 2020. Proteomics and metabolomics for cystic fibrosis research. Intl J Mol Sci 21:5439. doi:10.3390/ijms21155439 PMC7432297 32751630

[38] Pineau C. 2017. Looking for missing proteins in the testicular germ cell lineage: new insights into normal and pathological spermatogenesis. 17th C-HPP Symposium. Tehran, Iran

[39] Ohayon S, Girsault A, Nasser M, Shen-Orr S, Meller A. 2019. Simulation of single-protein nanopore sensing shows feasibility for whole-proteome identification. PLoS Comput Biol 15:e1007067. doi:10.1371/journal.pcbi.1007067 31145734 PMC6559672

[40] Palmblad M. 2021. Theoretical considerations for next-generation proteomics. J Proteome Res 20:3395–3399. doi:10.1021/acs.jproteome.1c00136 33904308 PMC8185883

[41] Swaminathan J, Boulgakov AA, Hernandez ET, Bardo AM, Bachman JL, Marotta J, Johnson AM, Anslyn EV, Marcotte EM. 2018. Highly parallel single-molecule identification of proteins in zeptomole-scale mixtures. Nat Biotechnol 10. doi:10.1038/nbt.4278 PMC6482110 30346938

[42] Kwon T, Huse HK, Vogel C, Whiteley M, Marcotte EM. 2014. Protein-to-mRNA ratios are conserved between Pseudomonas aeruginosa strains. J Proteome Res 13:2370–2380. doi:10.1021/pr4011684 24742327 PMC4012837

[43] Zhang M, Michie KL, Cornforth DM, Dolan SK, Wang Y, Whiteley M. 2023. Impact of growth rate on the protein-mRNA ratio in Pseudomonas aeruginosa. mBio 14:e03067-22. doi:10.1128/mbio.03067-22 36475772 PMC9973009

[44] Franks A, Airoldi E, Slavov N. 2017. Post-transcriptional regulation across human tissues. PLoS Comput Biol 13:e1005535. doi:10.1371/journal.pcbi.1005535 28481885 PMC5440056

[45] Edfors F, Danielsson F, Hallström BM, Käll L, Lundberg E, Pontén F, Forsström B, Uhlén M. 2016. Gene-specific correlation of RNA and protein levels in human cells and tissues. Mol Syst Biol 12:883. doi:10.15252/msb.20167144 27951527 PMC5081484

[46] Zhang C, Krause DJ, Whitaker RJ. 2013. Sulfolobus islandicus: a model system for evolutionary genomics. Biochem Soc Trans 41:458–462. doi:10.1042/BST20120338 23356328

[47] Zhang C, Phillips APR, Wipfler RL, Olsen GJ, Whitaker RJ. 2018. The essential genome of the crenarchaeal model Sulfolobus islandicus. Nat Commun 9:4908. doi:10.1038/s41467-018-07379-4 30464174 PMC6249222

[48] Zhang C, Cooper TE, Krause DJ, Whitaker RJ. 2013. Augmenting the genetic toolbox for Sulfolobus islandicus with a stringent positive selectable marker for agmatine prototrophy. Appl Environ Microbiol 79:5539–5549. doi:10.1128/AEM.01608-13 23835176 PMC3754178

[49] Poulsen BE, Yang R, Clatworthy AE, White T, Osmulski SJ, Li L, Penaranda C, Lander ES, Shoresh N, Hung DT. 2019. Defining the core essential genome of Pseudomonas aeruginosa. Proc Natl Acad Sci USA 116:10072–10080. doi:10.1073/pnas.1900570116 31036669 PMC6525520

[50] Turner KH, Wessel AK, Palmer GC, Murray JL, Whiteley M. 2015. Essential genome of Pseudomonas aeruginosa in cystic fibrosis sputum. Proc Natl Acad Sci USA 112:4110–4115. doi:10.1073/pnas.1419677112 25775563 PMC4386324

[51] Luo H, Lin Y, Liu T, Lai F-L, Zhang C-T, Gao F, Zhang R. 2021. DEG 15, an update of the Database of Essential Genes that includes built-in analysis tools. Nucleic Acids Res 49:D677–D686. doi:10.1093/nar/gkaa917 33095861 PMC7779065

[52] Zhang R, Ou HY, Zhang CT. 2004. DEG: a database of essential genes. Nucleic Acids Res 32:D271–D272. doi:10.1093/nar/gkh024 14681410 PMC308758

[53] Ji Y, Zhang B, Van SF, Warren P, Woodnutt G, Burnham MK, Rosenberg M, Horn. 2001. Identification of critical staphylococcal genes using conditional phenotypes generated by antisense RNA. Science 293:2266–2269. doi:10.1126/science.1063566 11567142

[54] Chaudhuri RR, Allen AG, Owen PJ, Shalom G, Stone K, Harrison M, Burgis TA, Lockyer M, Garcia-Lara J, Foster SJ, Pleasance SJ, Peters SE, Maskell DJ, Charles IG. 2009. Comprehensive identification of essential Staphylococcus aureus genes using Transposon-Mediated Differential Hybridisation (TMDH). BMC Genomics 10:291. doi:10.1186/1471-2164-10-291 19570206 PMC2721850

[55] Laurent JM, Vogel C, Kwon T, Craig SA, Boutz DR, Huse HK, Nozue K, Walia H, Whiteley M, Ronald PC, Marcotte EM. 2010. Protein abundances are more conserved than mRNA abundances across diverse taxa. Proteomics 10:4209–4212. doi:10.1002/pmic.201000327 21089048 PMC3113407

[56] Schrimpf SP, Weiss M, Reiter L, Ahrens CH, Jovanovic M, Malmström J, Brunner E, Mohanty S, Lercher MJ, Hunziker PE, Aebersold R, von Mering C, Hengartner MO. 2009. Comparative functional analysis of the Caenorhabditis elegans and Drosophila melanogaster proteomes. PLoS Biol 7:e48. doi:10.1371/journal.pbio.1000048 19260763 PMC2650730

[57] Vogel C, Marcotte EM. 2012. Insights into the regulation of protein abundance from proteomic and transcriptomic analyses. Nat Rev Genet 13:227–232. doi:10.1038/nrg3185 22411467 PMC3654667

[58] Chen H, Zhang Z, Jiang S, Li R, Li W, Zhao C, Hong H, Huang X, Li H, Bo X. 2020. New insights on human essential genes based on integrated analysis and the construction of the HEGIAP web-based platform. Brief Bioinform 21:1397–1410. doi:10.1093/bib/bbz072 31504171 PMC7373178

[59] Deutschbauer AM, Jaramillo DF, Proctor M, Kumm J, Hillenmeyer ME, Davis RW, Nislow C, Giaever G. 2005. Mechanisms of haploinsufficiency revealed by genome-wide profiling in yeast. Genetics 169:1915–1925. doi:10.1534/genetics.104.036871 15716499 PMC1449596

[60] Belda-Ferre P, Williamson J, Simón-Soro Á, Artacho A, Jensen ON, Mira A. 2015. The human oral metaproteome reveals potential biomarkers for caries disease. Proteomics 15:3497–3507. doi:10.1002/pmic.201400600 26272225

[61] Wu X, Siehnel RJ, Garudathri J, Staudinger BJ, Hisert KB, Ozer EA, Hauser AR, Eng JK, Manoil C, Singh PK, Bruce JE. 2019. In vivo proteome of Pseudomonas aeruginosa in airways of cystic fibrosis patients. J Proteome Res 18:2601–2612. doi:10.1021/acs.jproteome.9b00122 31060355 PMC6750005

[62] Jorth P, Whiteley M. 2010. Characterization of a novel riboswitch-regulated lysine transporter in Aggregatibacter actinomycetemcomitans. J Bacteriol 192:6240–6250. doi:10.1128/JB.00935-10 20889741 PMC2981213

[63] Lewin GR, Stacy A, Michie KL, Lamont RJ, Whiteley M. 2019. Large-scale identification of pathogen essential genes during coinfection with sympatric and allopatric microbes. Proc Natl Acad Sci USA 116:19685–19694. doi:10.1073/pnas.1907619116 31427504 PMC6765283

[64] Whiteley M. 2001. Quorum sensing and biofilm development in Pseudomonas aeruginosa Doctoral thesis, Microbiology University of Iowa

[65] Whiteley M, Ott JR, Weaver EA, McLean RJ. 2001. Effects of community composition and growth rate on aquifer biofilm bacteria and their susceptibility to betadine disinfection. Environ Microbiol 3:43–52. doi:10.1046/j.1462-2920.2001.00158.x 11225722

[66] Whiteley M, Bangera MG, Bumgarner RE, Parsek MR, Teitzel GM, Lory S, Greenberg EP. 2001. Gene expression in Pseudomonas aeruginosa biofilms. Nature 413:860–864. doi:10.1038/35101627 11677611

[67] Higginbotham L, Ping L, Dammer EB, Duong DM, Zhou M, Gearing M, Hurst C, Glass JD, Factor SA, Johnson ECB, Hajjar I, Lah JJ, Levey AI, Seyfried NT. 2020. Integrated proteomics reveals brain-based cerebrospinal fluid biomarkers in asymptomatic and symptomatic Alzheimer’s disease. Sci Adv 6:eaaz9360. doi:10.1126/sciadv.aaz9360 33087358 PMC7577712

[68] Zlatic SA, Duong D, Gadalla KKE, Murage B, Ping L, Shah R, Fink JJ, Khwaja O, Swanson LC, Sahin M, Rayaprolu S, Kumar P, Rangaraju S, Bird A, Tarquinio D, Carpenter R, Cobb S, Faundez V. 2022. Convergent cerebrospinal fluid proteomes and metabolic ontologies in humans and animal models of Rett syndrome. iScience 25:104966. doi:10.1016/j.isci.2022.104966 36060065 PMC9437849

[69] Emms DM, Kelly S. 2015. OrthoFinder: solving fundamental biases in whole genome comparisons dramatically improves orthogroup inference accuracy. Genome Biol 16:157. doi:10.1186/s13059-015-0721-2 26243257 PMC4531804

[70] Emms DM, Kelly S. 2019. OrthoFinder: phylogenetic orthology inference for comparative genomics. Genome Biol 20:238. doi:10.1186/s13059-019-1832-y 31727128 PMC6857279

[71] Altschul SF, Gish W, Miller W, Myers EW, Lipman DJ. 1990. Basic local alignment search tool. J Mol Biol 215:403–410. doi:10.1016/S0022-2836(05)80360-2 2231712

[72] Buchfink B, Xie C, Huson DH. 2015. Fast and sensitive protein alignment using DIAMOND. Nat Methods 12:59–60. doi:10.1038/nmeth.3176 25402007

