# Supplementary material for: Conserved cross-domain protein-to-mRNA ratios enable proteome prediction in microbes
Source: mBio. 2025 Jul 24;16(8):e01411-25. doi: 10.1128/mbio.01411-25 (PMC12345168; doi:10.1128/mbio.01411-25)
Supplement: Supplemental figures — Figures S1 to S6. [file mbio.01411-25-s0005.pdf]

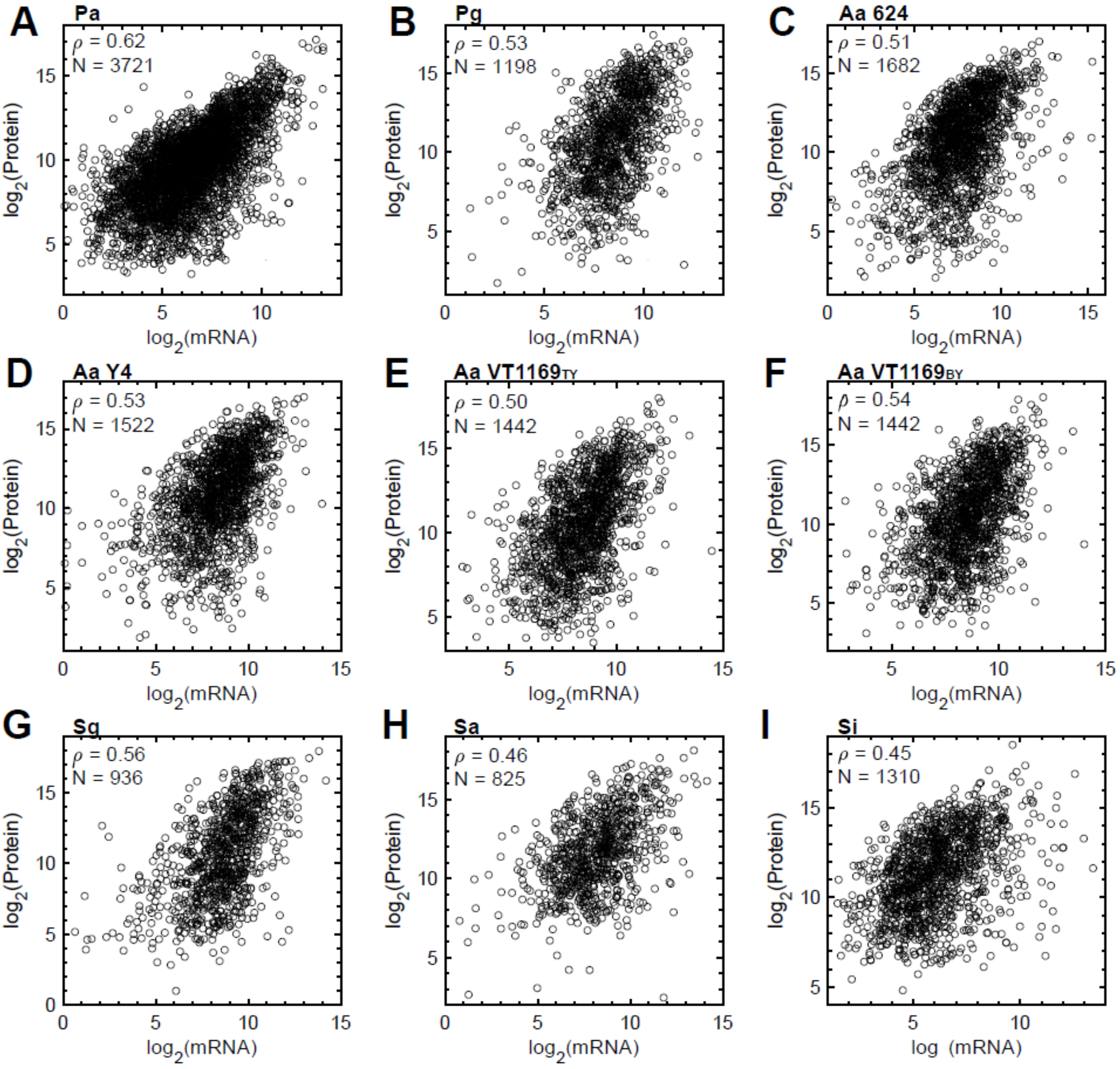

Fig. S1: Correlation between mRNA and protein levels across diverse microbes. Scatterplots shows the relationship between protein and mRNA abundances across diverse taxa, encompassing Gram-negative bacteria (A-F), Gram-positive bacteria (G-H) and archaea (I). Spearman rank correlation coefficients (ρ) were employed to assess the association. Genes included were detected in both transcriptome and proteome datasets.

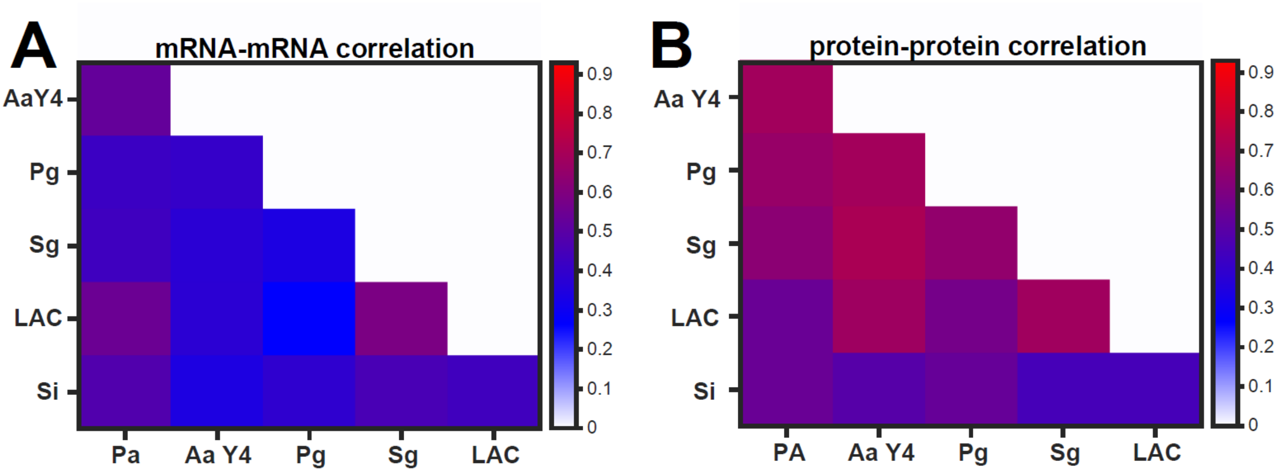

Fig. S2: Subsampling analysis including only one *A. actinomycetemcomitans* species (Y4). Assessment of the pairwise correlations between mRNA or protein levels using subsampled datasets with only one *A. actinomycetemcomitans* species (Y4). p-values were calculated using Wilcoxon signed rank test ($p < 10^{-4}$).

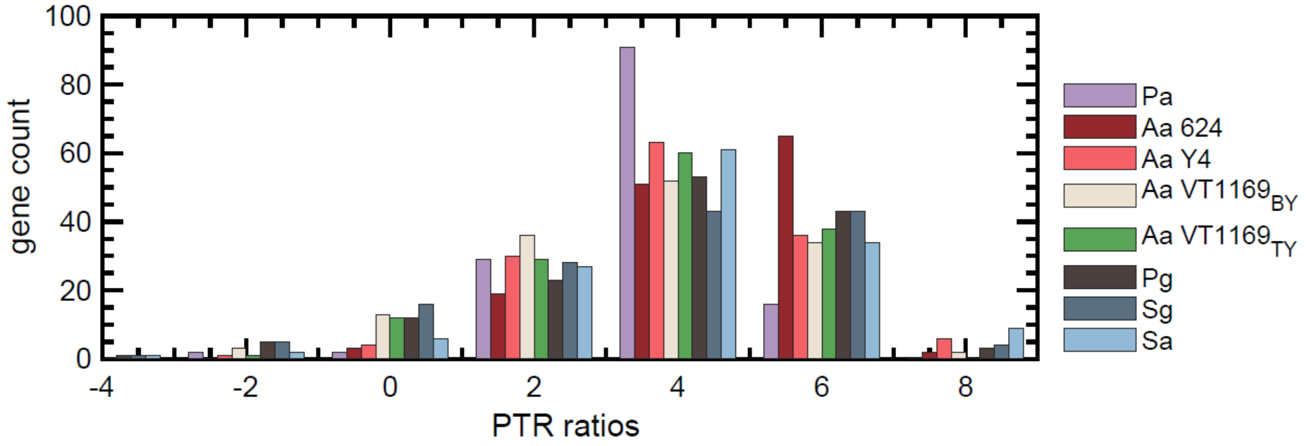

Fig. S3. Distribution of ptr ratios across microbes. The ptr ratios displayed a 'left-skewed' distribution for orthologous genes across all bacterial species, indicating an increased prevalence of lower ptr values.

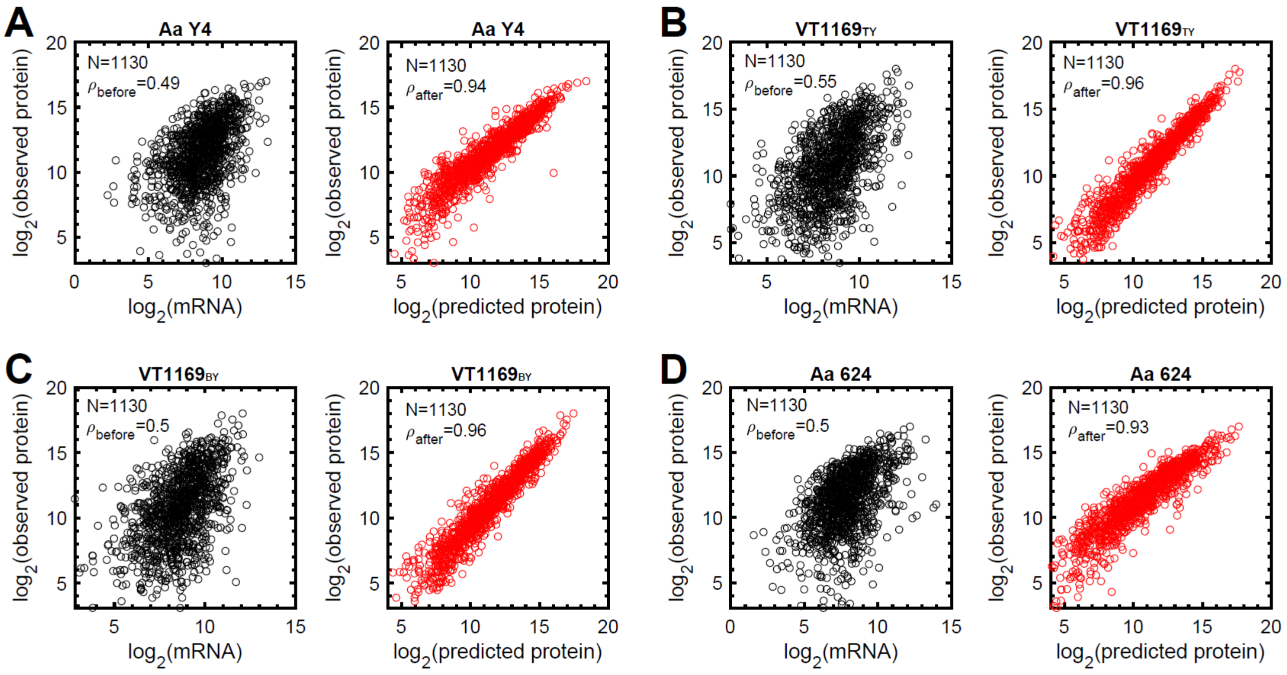

Fig. S4: RTP conversion factors calculated from three A*. actinomycetemcomitans* (*Aa*) datasets improves the predictivity of protein levels from mRNA abundances in the left out dataset. Improved predictivity was observed for **(A)** *Aa* Y4, **(B)** *Aa* $VT1169_{TY}$, **(C)** *Aa* $VT1169_{BY}$, and **(D)** *Aa* 624. As an example, for *Aa* Y4 **(A)** RTP conversion factors were calculated using data from the three other *Aa* strains ($VT1169_{TY}$, $VT1169_{BY}$, and *Aa* 624), then applied to Y4 datasets. Spearman rank correlation coefficients were calculated before (ρ_{before}, left graph black) and after (ρ_{after}, right graph red) application of the RTP conversion factors. N is the number of genes used.

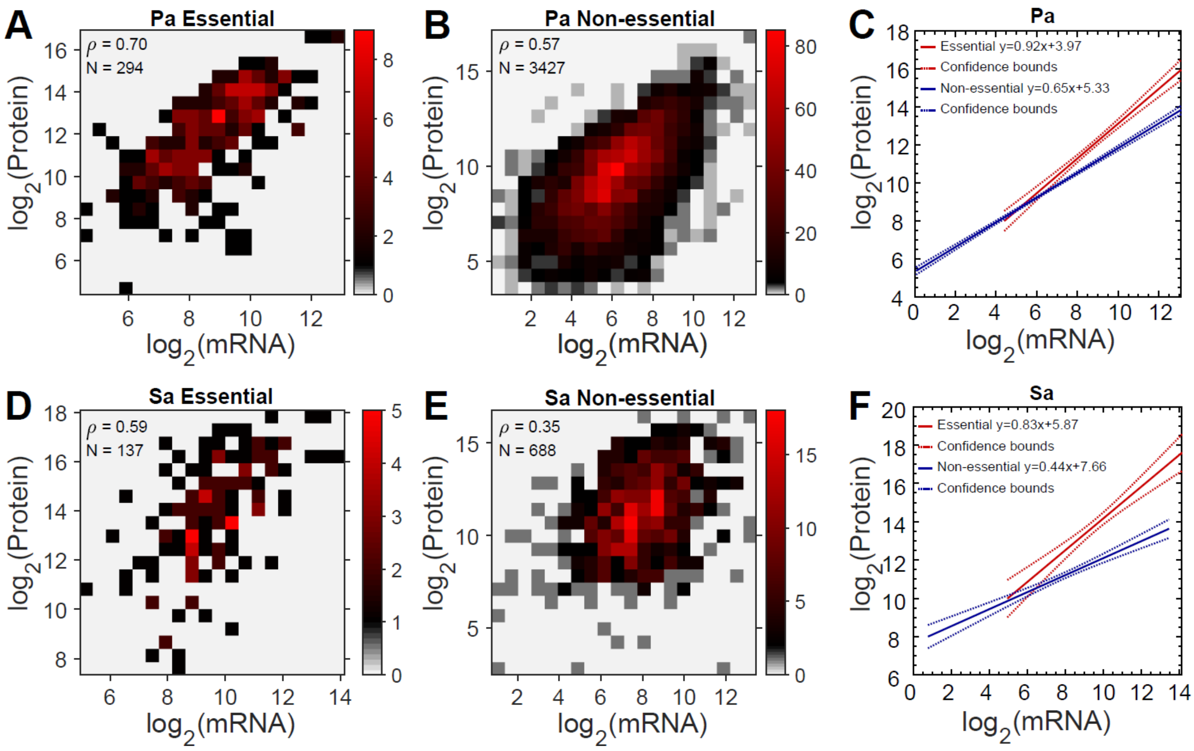

Figure S5: Density scatterplot and regression analysis of essential and nonessential genes. Binned scatterplots showing the relationship between measured protein abundances and mRNA abundances in *P. aeruginosa* (A-B) and *S. aureus* (D-E). The numbers of genes within each correlation are represented by the color scale in the right ordinate, where the number indicates the number of genes at a specific x-y coordinate. Best-fit regression lines for essential and nonessential genes with associated 95% confidence bounds in *P. aeruginosa* (C) and *S. aureus* (F). Essential genes exhibit a steeper slope than nonessential genes in both species. p-values were calculated using an F-test of overall significance ($p < 10^{-39}$ for *P. aeruginosa*, $p < 10^{-12}$ for *S. aureus*).

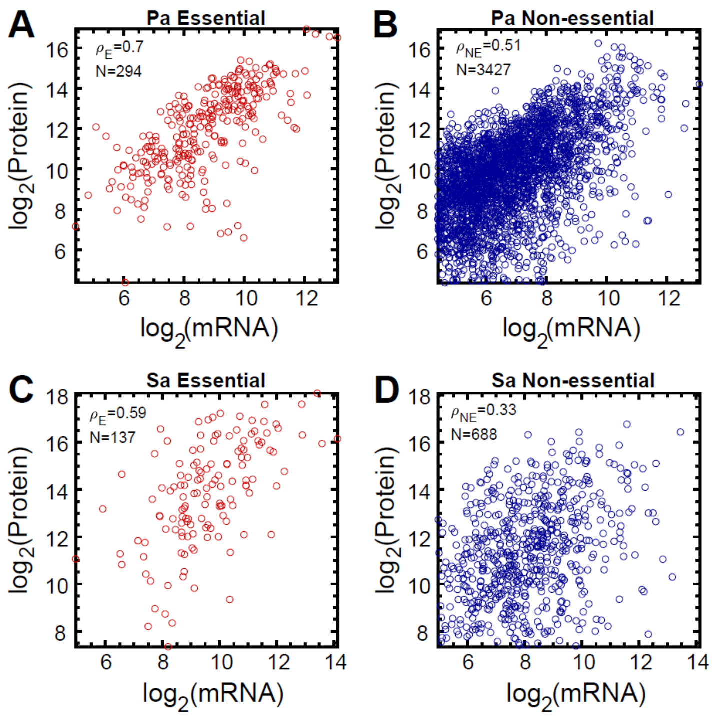

Figure S6: Higher correlation between mRNA and protein abundances for essential genes compared to nonessential genes is not a result of higher expression levels of essential genes. Scatterplot of mRNA and protein abundances in *P. aeruginosa* (**A-B**) and *S. aureus* (**C-D**) with Spearman rank correlation coefficients for essential genes and nonessential genes, limited to genes with comparable expression levels. N is the number of genes used.
